# Mcu regulates bone formation via mitochondrial calcium uptake and lineage allocation

**DOI:** 10.1038/s12276-026-01705-3

**Published:** 2026-05-01

**Authors:** Suji Kim, Hanna Jeong, Tue Nguyen Hoang, Kyu-sang Park, Jun Namkung

**Affiliations:** 1 Organelle Medicine Research Center, Yonsei University Wonju College of Medicine, Wonju, Republic of Korea; 2 Department of Global Medical Science, Yonsei University Wonju College of Medicine, Wonju, Republic of Korea; 3 Department of Biochemistry, Yonsei University Wonju College of Medicine, Wonju, Republic of Korea; 4 Institute of Mitochondrial Medicine, Yonsei University Wonju College of Medicine, Wonju, Republic of Korea; 5 Department of Physiology, Yonsei University Wonju College of Medicine, Wonju, Republic of Korea

**Keywords:** Bone remodelling, Mesenchymal stem cells, Bone

## Abstract

The mitochondrial calcium uniporter (Mcu) mediates calcium influx into the mitochondrial matrix, playing an essential role in cellular energy metabolism and survival. Although Mcu has been studied in various physiological contexts, its role in skeletal homeostasis remains poorly understood. Here we investigate how *Mcu* deficiency affects osteoblast differentiation and bone formation under aging-related stress. Using an inducible whole-body *Mcu*-knockout mouse model, we found that *Mcu* deletion resulted in impaired mitochondrial calcium uptake, reduced oxidative phosphorylation, fragmented mitochondrial morphology and decreased expression of osteogenic genes, leading to defective osteogenesis. Concurrently, adipogenic markers were elevated in *Mcu*-deficient bone marrow cells, indicating altered mesenchymal lineage commitment. Mechanistically, *Mcu*-deficient cells exhibited enhanced TGF-β signaling and reduced BMP/Wnt pathway activity. In vivo, inducible whole-body *Mcu*-knockout mice exhibited reduced trabecular bone volume and density while maintaining normal skeletal growth. Pharmacological modulation of mitochondrial calcium influx using kaempferol enhanced osteogenic differentiation and mitochondrial respiration in wild-type, but not *Mcu*-deficient, cells. Consistently, analysis of publicly available human datasets revealed age- and osteoporosis-associated downregulation of *MCU* expression in bone tissues. These findings suggest that Mcu regulates bone formation by controlling mitochondrial calcium uptake and mesenchymal lineage allocation. Targeting mitochondrial calcium signaling may offer novel therapeutic strategies for age-related skeletal disorders.

## Targeting mitochondrial calcium signaling for bone disorders

This study explores the role of the mitochondrial calcium uniporter (MCU) in bone health, focusing on its impact on osteoblast differentiation and bone integrity. Researchers used an inducible whole-body *Mcu*-knockout mouse model to investigate how MCU deficiency affects bone formation and mesenchymal lineage allocation. They found that Mcu deficiency impairs mitochondrial respiration, reduces osteogenic potential and increases adipogenesis in bone marrow-derived cells, leading to diminished bone formation and increased marrow adiposity in aged mice. Results showed that Mcu is crucial for maintaining mitochondrial bioenergetics and dynamics during osteoblast differentiation, linking mitochondrial calcium signaling to bone biology. The study suggests that pharmacological modulation of mitochondrial calcium uptake, for example with kaempferol, could enhance osteogenesis and may offer potential therapeutic strategies for bone loss disorders.

This summary was initially drafted using artificial intelligence, then revised and fact-checked by the author.

## Introduction

Bone undergoes continuous remodeling through a balanced interplay between osteoblast-driven bone formation and osteoclast-mediated resorption to maintain structural integrity and function^1^. Osteoblasts, differentiated from mesenchymal stem cells, are responsible for matrix synthesis and mineralization—an energy-intensive process that heavily relies on mitochondrial metabolism^2–4^. Recent work emphasizes that cellular metabolism and signaling significantly influence osteoblast differentiation and overall bone health^5^.

Mitochondria exert critical roles in osteoblasts by supplying ATP through oxidative phosphorylation (OXPHOS) and coordinating anabolic process necessary for differentiation^6^. Indeed, mitochondrial content and function changes are tightly linked to bone cell fate, coupling metabolic capacity with lineage specification^6,7^. However, the molecular mechanisms that govern mitochondrial adaptation in osteoblasts remain incompletely understood.

Calcium, a key second messenger, integrates cell signaling and metabolism. In bone, calcium modulates critical differentiation pathways such as BMP/Wnt and TGF-β^8,9^. Beyond cytosolic signaling, mitochondrial calcium uptake regulates metabolic shifts from glycolysis to OXPHOS^10^. This mitochondrial uptake occurs primarily through the mitochondrial calcium uniporter (MCU) complex^11,12^ and directly enhances key metabolic enzymes such as pyruvate dehydrogenase and oxoglutarate dehydrogenase, thereby influencing ATP synthesis, reactive oxygen species (ROS) production and apoptosis^13–15^. Studies in muscle and cardiac tissues demonstrate that Mcu-mediated calcium uptake influences metabolic fuel selection and energy homeostasis^16,17^, suggesting similar roles in osteoblast differentiation.

Although MCU-dependent mitochondrial calcium uptake is known to regulate mitochondrial dynamics, redox status and transcriptional control in various cell types^18,19,11^, its role in skeletal homeostasis—particularly under age-related stress—is not thoroughly explored. We hypothesized that *Mcu* deficiency impairs osteoblast function by disrupting mitochondrial bioenergetics, thereby altering mesenchymal lineage allocation.

To test this, we used an inducible whole-body *Mcu*-knockout (*Mcu* iKO) mouse model that bypass early developmental lethality in C57BL/6 background^20^, enabling analysis of skeletal outcomes in aging mice over 40 weeks. Our results demonstrate impaired mitochondrial respiration, reduced osteogenic potential and increased adipogenesis in *Mcu*-deficient bone marrow-derived cells, accompanied by disrupted mitochondrial dynamics and diminished bone formation in vivo. Furthermore, pharmacologic modulation of mitochondrial calcium uptake with kaempferol supported these findings. Collectively, our study identifies Mcu as a critical regulator of mitochondrial metabolism and mesenchymal lineage fate, offering new insights into the role of mitochondrial calcium signaling in bone biology and skeletal aging.

## Materials and methods

### Mice

All mouse experiments were conducted under protocols approved by the Institutional Animal Care and Use Committee at Yonsei University Wonju College of Medicine (approval number YWC-181022-1). *Mcu* floxed mice (The Jackson Laboratory #029817) were crossed with *Rosa26Cre-ERT2* mice (The Jackson Laboratory #008463). Tamoxifen (T5648, Sigma) was dissolved in corn oil to a working concentration of 20 mg/ml. To induce Cre recombination-mediated deletion of *Mcu*, tamoxifen was treated at a dosage of 75 mg/kg body weight via intraperitoneal injection daily for 5 consecutive days, starting at 6 weeks of age. Tamoxifen-administered *Mcu*^*flox/flox*^; *Rosa26Cre-ERT2*^*Tg/Wt*^ mice were compared with *Mcu*^*flox/flox*^ littermate controls. For ovariectomy experiments, bilateral ovariectomy was performed on 5-week-old female mice, as previously described^21^. Sham-operated mice underwent the same procedure without ovary removal. Mice were euthanized and femurs were collected 25 weeks after surgery. All mice used in this study were male, except those in the ovariectomy group.

### μCT and quantification

Femurs were collected from the experimental mice at 10 months of age and fixed in 4% paraformaldehyde dissolved in phosphate-buffered saline (PBS) for 18 h at 4 °C. The fixed samples were then washed with PBS. Micro-computed tomography (μCT) imaging was performed using a Quantum GX2 microCT (PerkinElmer) under the following conditions: 90 kV voltage, 88 μA current, 10 μm voxel size. The acquired images were analyzed and quantified using Quantum Gx software.

### Urine and serum biochemical analyses

Urine and serum biochemical analyses were performed using samples collected from 40-week-old mice. Urine samples were obtained by placing mice individually in clean collection containers. For serum preparation, mice were anesthetized by inhalation anesthesia, and whole blood was collected via orbital enucleation using serum collection tubes. Blood samples were allowed to clot at room temperature and then centrifuged at 2,000*g* for 10 min. Urinary calcium and phosphate concentrations were measured using commercially available assay kits (SA10036300, JW: 309394-005, Sekisui). Serum levels of Fgf23, parathyroid hormone and vitamin D3 were quantified using a commercial immunoassay kit (EM0271 and EM1319, FineTest; MBS731507, Mybiosource). All assays were performed according to the manufacturers’ instructions, and concentrations were determined on the basis of standard curves provided with each kit.

### Cell culture

MC3T3-E1 pre-osteoblast cells, C3H10T1/2 cells and bone marrow cells (BMCs) isolated from the femurs of *Mcu*^flox/flox^; *Rosa26Cre-ERT2*^*Tg/Wt*^ mice were cultured and differentiated in vitro. BMCs are isolated by flushing the bone marrow out of the femurs using a needle and sterile PBS. The flushed marrow is then passed through a cell strainer to obtain a cell suspension. All cells were maintained in Dulbecco’s modified Eagle medium (Gibco) supplemented with 10% fetal bovine serum and 1% penicillin–streptomycin. For osteogenic differentiation, cells were treated with osteogenic medium containing 10 mM β-glycerophosphate (Sigma) and 50 μg/ml ascorbic acid (Sigma) with α-MEM. Differentiation was carried out for 7–14 days, with medium changes every 2–3 days. In the case of C3H10T1/2 cells and BMCs, adipogenesis was induced using differentiation medium consisting of high-glucose Dulbecco’s modified Eagle medium supplemented with 0.5 mM isobutylmethylxanthine (Sigma), 1 μM dexamethasone and 1 μg/ml insulin. Cells were differentiated for 5–7 days, and the medium was refreshed every 3 days. At the end of the differentiation period, cells were collected for RNA and protein extraction. For indirect co-culture, MC3T3-E1 cells were seeded in the bottom chamber plates, while *Mcu* wild-type (WT) or iKO BMCs were placed in 0.4-μm-pore Transwell inserts (Corning). Cells were co-cultured in osteogenic medium for 7–10 days. Cells were transfected with small interfering RNA (siRNA) targeting *Mcu* or nontargeting control using Lipofectamine RNAiMAX (Invitrogen) according to the manufacturer’s protocol. Cells were seeded in a six-well plate and transfected at approximately 80–90% confluency with 30–50 nM siRNA. After 6 h, the medium was replaced with complete growth medium. Cells were cultured for 48 h after transfection before being collected for experiments.

### Real-time PCR

Total RNA was isolated using the TRIzol reagent (Invitrogen) following the manufacturer’s instructions. Reverse transcription was performed using a SuperScript III reverse transcription kit (Thermo Fisher Scientific). Quantitative polymerase chain reaction (qPCR) was conducted on the Quant-Studio 6 Real-Time PCR System (Thermo Fisher Scientific). *Rplp0* was used for normalization of the relative expression of target genes. Mitochondrial DNA copy number was quantified by qPCR using genomic DNA, with *mt-Nd6* and *Gapdh* as mitochondrial and nuclear targets, respectively. The primer sequences used for PCR are presented in Supplementary Table 1.

### Western blotting

Proteins were extracted by homogenizing the samples in ice-cold RIPA lysis buffer supplemented with protease and phosphatase inhibitors (Thermo Fisher Scientific). Protein concentrations were measured using the BCA Protein Assay Kit (Thermo Fisher Scientific). Protein samples were resolved on sodium dodecyl sulfate–polyacrylamide gel electrophoresis gels and transferred to polyvinylidene difluoride membranes using a wet transfer system (Bio-Rad) according to the manufacturer’s protocol. Membranes were blocked with 5% skim milk for 1 h to prevent nonspecific binding. After blocking, the membranes were incubated overnight at 4 °C with primary antibodies specific to the proteins of interest. Protein bands were detected using an enhanced chemiluminescence reagent (Cytiva Amersham) and visualized with a ChemiDoc Imaging System (Bio-Rad). Quantification of protein levels was performed by normalizing target protein signals to the β-actin. Antibodies used in western blotting are listed in Supplementary Table 2.

### Mitochondrial calcium uptake assay

After cell culture, cells were washed twice with calcium-free Krebs–Ringer bicarbonate buffer (KRBB). Mitochondria were isolated using the Dounce homogenization method with a commercial isolation kit (Thermo Scientific), following the manufacturer’s instructions. A staining solution containing 100 nM Calcium Green-5N (Invitrogen) in calcium-free KRBB was applied to the cells, followed by incubation at 37 °C for 10 min. After staining, the cells were washed twice with PBS to remove excess dye, and calcium-free KRBB was added. Fluorescence intensity was measured using a FlexStation 3 (Molecular Devices) and fluorescence microscope (Leica) with excitation and emission wavelengths of 490 nm and 516 nm, respectively. Mitochondrial calcium uptake was assessed by monitoring changes in fluorescence intensity.

### Histological analysis

For cell-based assays, MC3T3-E1 cells or bone marrow-derived cells were subjected to the following protocols. Alkaline phosphatase (ALP) staining was performed after fixation with 4% paraformaldehyde for 15 min, using an ALP staining kit (Sigma) according to the manufacturer’s instructions, to assess early osteogenic activity. Alizarin Red S staining (2%, pH 4.2) and von Kossa staining (5% silver nitrate under ultraviolet light) were used to visualize matrix mineralization. For Oil Red O staining, cells were fixed in 4% paraformaldehyde and stained with Oil Red O solution to detect lipid droplets. For tissue analysis, femurs were fixed in 10% formalin, embedded in paraffin and sectioned at a thickness of 8 μm. Hematoxylin and eosin (H&E) staining was then performed to evaluate bone marrow morphology, adipocyte accumulation and kidney structures.

### Measurement of mitochondrial membrane potential

JC-1 dye (Invitrogen) was prepared at a final concentration of 1 µM in KRBB and applied to the cells. The cells were incubated with the JC-1 solution at 37 °C for 30 min. After incubation, the JC-1 solution was removed, and the cells were washed twice with KRBB to eliminate excess dye. Fluorescence intensity was measured using a fluorescence microscope or plate reader with excitation/emission wavelengths of 490/530 nm for the JC-1 monomers (green fluorescence) and 540/590 nm for the JC-1 aggregates (red fluorescence). The mitochondrial membrane potential was evaluated by calculating the ratio of red to green fluorescence intensity.

### TEM

Cells were fixed in 2.5% glutaraldehyde in 0.1 M phosphate buffer (pH 7.4) at 4 °C for 24 h. The samples were postfixed in 1% osmium tetroxide, dehydrated through a graded ethanol series and embedded in epoxy resin. Ultrathin sections (~70 nm) were cut using an ultramicrotome and mounted on copper grids. Sections were stained with uranyl acetate and lead citrate, and mitochondria were observed using a transmission electron microscope (HT7800, Hitachi) at an accelerating voltage of 80 kV.

### OCR measurement

The oxygen consumption rate (OCR) in cultured cells was measured using the Seahorse XF Analyzer (Agilent) in a 96-well format. After differentiation, cells were incubated in Seahorse XF Assay Medium. Oligomycin, FCCP and rotenone/antimycin A (all from Agilent) were sequentially injected to determine basal respiration, maximal respiration, ATP production and proton leak according to standard calculations.

### RNA sequencing analysis of human bone biopsies

To evaluate MCU expression in human bone tissues, we analyzed the publicly available dataset GSE72815 from the Gene Expression Omnibus, which includes gene expression profiles of iliac crest bone biopsies from postmenopausal women with and without osteoporosis.

### Statistics

All data are expressed as mean ± standard error of the mean (s.e.m.). Statistical analyses were conducted using GraphPad Prism (version 10). For group comparisons, an unpaired two-tailed Student’s *t*-test or one-way analysis of variance (ANOVA) was applied. Statistical significance was defined as *P < 0.05, **P < 0.01, ***P < 0.001, ****P < 0.0001.

## Results

### Aging impairs osteogenic differentiation in BMCs

**Fig. 1:**
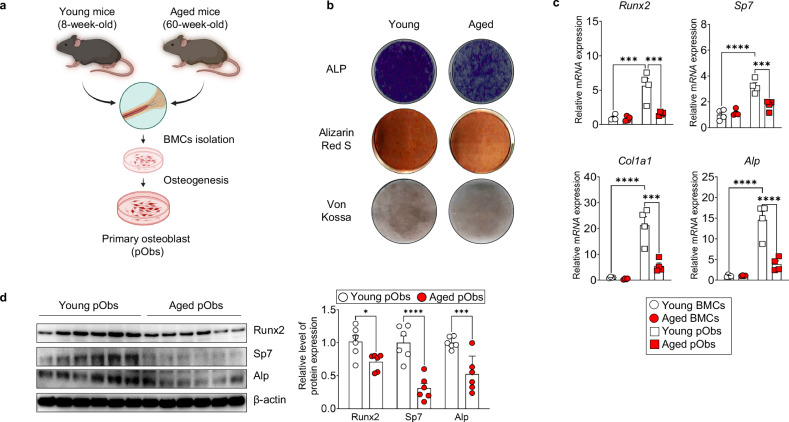
Reduced osteogenic differentiation capacity in BMC-derived osteoblasts from aged mice. **a** A schematic illustration of the experimental procedure. BMCs were isolated from femurs of either 8-week-old or 60-week-old mice and cultured in osteogenic medium to induce differentiation into pObs. **b** Representative images of histochemical staining in pObs derived from young and aged mice. **c** Quantitative real-time PCR analysis of osteogenic marker expression in pObs from young and aged mice. **d** Immunoblot analysis of osteogenic markers in pObs. Data are presented as mean ± s.e.m. Statistical significance: *P < 0.05, ***P < 0.001, ****P < 0.0001.

To evaluate the impact of aging on osteogenic potential of BMCs, we compared primary osteoblasts (pObs) isolated from young (8-week-old) and aged (60-week-old) mice (Fig. 1a). ALP, Alizarin Red S and von Kossa staining revealed reduced mineralization in aged pObs, indicating impaired osteogenic differentiation (Fig. 1b). Expression analysis confirmed significantly lower levels of osteogenic markers in aged BMCs under differentiation conditions (Fig. 1c, d). In contrast to a decline in osteogenesis, aged BMCs exhibited upregulation of adipogenic genes under adipogenic conditions (Supplementary Fig. 1), suggesting that aging may skew mesenchymal differentiation potential toward adipogenesis. Collectively, these findings demonstrate that aging impairs the osteogenic differentiation capacity of BMC-derived osteoblasts.

### *Mcu* supports osteogenic differentiation via mitochondrial calcium uptake

Building on the finding that aging impairs osteogenic differentiation (Fig. 1), we next investigated whether mitochondrial changes contribute to this defect. We first observed a reduction in mitochondrial proteins in osteoblasts derived from aged BMCs (Supplementary Fig. 2a). Given that Mcu governs calcium-dependent mitochondrial bioenergetics, we hypothesized that age-related downregulation of Mcu might underlie this impairment. Consistent with this idea, analysis of publicly available human data revealed significantly reduced MCU expression in elderly women with osteoporosis^22^ (Supplementary Fig. 2b). We further validated this finding in both aged mice (Supplementary Fig. 2c) and ovariectomized mice, a model of postmenopausal osteoporosis (Supplementary Fig. 2d). Together, these findings suggest that age-related skeletal stress may reduce Mcu expression in bone tissues, potentially compromising mitochondrial calcium handling during osteogenesis.

**Fig. 2:**
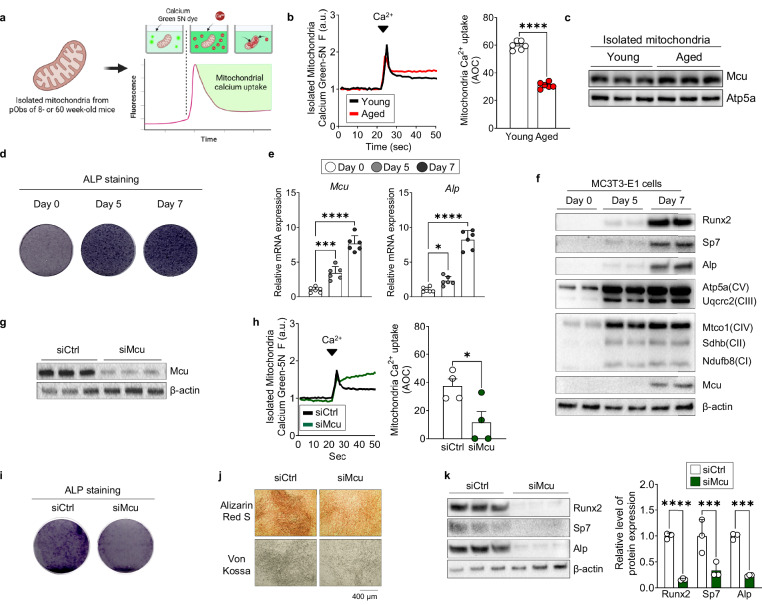
Impaired mitochondrial calcium uptake, due to aging or *Mcu* loss, suppresses osteogenic differentiation. **a** Schematic overview of mitochondrial calcium uptake measurement from mitochondrial isolation from pObs derived from young (8-week) or aged (60-week) mice. **b** Mitochondrial calcium uptake measured using Calcium Green-5N in isolated mitochondria treated with 5 μM calcium. Representative traces and quantification shown. **c** Immunoblot analysis of mitochondrial proteins in equal amounts of mitochondria isolated from each group. **d** ALP staining in MC3T3-E1 cells during osteogenic differentiation. **e** qPCR analysis of mitochondrial and osteogenic gene expression across different time points. **f** Immunoblot of OXPHOS subunits and osteogenic markers during MC3T3-E1 differentiation. **g** siRNA-mediated knockdown efficiency of *Mcu* confirmed by immunoblot. **h** Mitochondrial calcium uptake in isolated mitochondria from siRNA-treated cells. **i** Representative ALP staining on day 7 post-knockdown. **j** Von Kossa and Alizarin Red S staining after 14 days of differentiation. **k** Immunoblot analysis of osteogenic markers on day 7 post-knockdown. Data are presented as mean ± s.e.m. Statistical significance: *P < 0.05, ***P < 0.001, ****P < 0.0001.

To directly test whether mitochondrial calcium uptake is impaired with age, we isolated mitochondria from femurs of young and aged mice and measured calcium uptake capacity (Fig. 2a). Aged mitochondria showed significantly reduced calcium uptake (Fig. 2b). Interestingly, Mcu protein levels normalized to mitochondrial mass were comparable (Fig. 2c), suggesting that post-translational regulation or impaired activity due to altered membrane potential or interaction with binding partners may contribute to functional differences. To evaluate the role of Mcu during osteoblast differentiation, we examined its expression in MC3T3-E1 cells during differentiation. ALP staining confirmed progressive osteoblast maturation (Fig. 2d). Both *Mcu* mRNA and protein levels increased over time, indicating a correlation with differentiation progression (Fig. 2e, f and Supplementary Fig. 3a). To determine the functional requirement of Mcu, we silenced its expression using siRNA (Fig. 2g). *Mcu* knockdown significantly reduced mitochondrial calcium uptake (Fig. 2h) and impaired osteoblast differentiation, as shown by decreased ALP activity and matrix mineralization (Fig. 2i, j). Osteogenic gene expression was also markedly reduced in *Mcu*-deficient cells (Fig. 2k and Supplementary Fig. 3b). Together, these findings demonstrate that Mcu-mediated mitochondrial calcium uptake is essential for sustaining osteoblast differentiation and matrix mineralization, thereby linking mitochondrial bioenergetics to mesenchymal lineage specification.

### *Mcu* deletion leads to reduced bone mass and marrow adiposity in aged mice

**Fig. 3:**
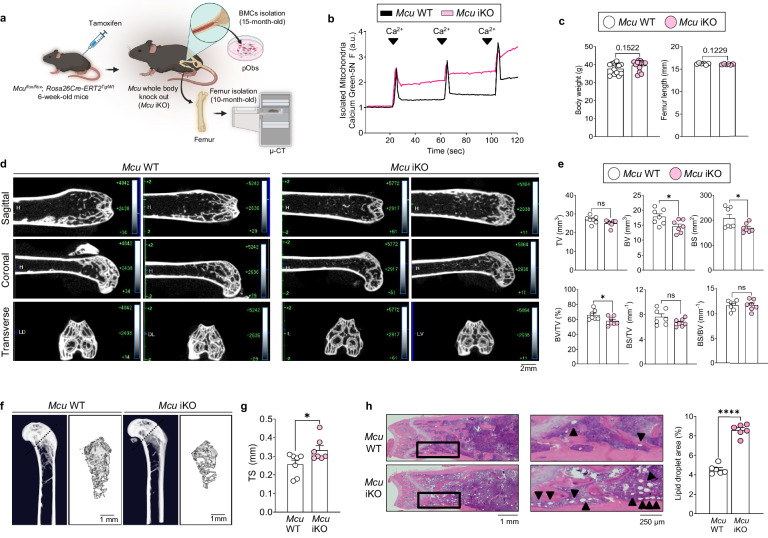
*Mcu* deletion impairs bone structure and promotes marrow adiposity in aged mice. **a** Schematic of inducible *Mcu*-knockout model. Tamoxifen was administered to 6-week-old male mice, and analysis was conducted at 40–60 weeks. **b** Mitochondrial calcium uptake assessed in isolated mitochondria from femurs of *Mcu* iKO and control mice using Calcium Green-5N. **c** Body weight and femur length measurements at 10 months of age. **d** Representative μCT images of femurs. **e** μCT-based morphometric analysis. **f** Three-dimensional reconstruction of trabecular structure in femoral neck. **g** Quantification of trabecular separation. **h** H&E staining of femoral marrow. All experiments were performed in mice aged 40 weeks. TV, total volume; BV, bone volume; BS, bone surface; TS, trabecular separation. Data are presented as mean ± s.e.m. Statistical significance: *P < 0.05, ****P < 0.0001.

These in vitro findings prompted us to investigate whether *Mcu* deficiency alters bone structure and mesenchymal lineage allocation in vivo. To this end, we generated an inducible *Mcu* knockout (*Mcu* iKO) mouse model using a tamoxifen-inducible Cre system (Fig. 3a). Functional deletion of Mcu was confirmed by a marked reduction in mitochondrial calcium uptake (Fig. 3b) and decreased phosphorylation of pyruvate dehydrogenase (Supplementary Fig. 4a). To determine whether Mcu is required for normal skeletal development, we first evaluated body weight and bone length in 10-month-old mice. There were no significant differences in body weight, tibia length or femur length between *Mcu* iKO and control mice (Fig. 3c and Supplementary Fig. 4b), indicating that *Mcu* is not essential for gross skeletal growth per se. However, μCT revealed substantial architectural deterioration in both cortical and trabecular compartments of *Mcu* iKO femurs. Quantitative analysis showed reductions in total bone volume, bone surface area, bone volume fraction, and cortical and trabecular bone mineral density, along with increased trabecular separation (Fig. 3d–g and Supplementary Fig. 4c). Three-dimensional μCT reconstructions further illustrated the loss of trabecular integrity in femoral neck (Fig. 3f and Supplementary Fig. 4d). To assess potential shifts in bone marrow composition, we performed histological analysis of femoral sections from aged *Mcu* iKO mice. H&E staining revealed a significant increase in marrow adipocytes in *Mcu* iKO mice compared with controls (Fig. 3h). These findings suggest that *Mcu* deficiency in osteogenic lineage cells not only impairs bone mass maintenance but also promotes mesenchymal commitment toward adipogenesis in vivo. Given the prominent marrow adiposity and bone loss observed in aged *Mcu* iKO mice, we next evaluated whether these skeletal phenotypes might be influenced by classical renal–endocrine mineral homeostasis. To address this, we examined renal histology and key endocrine parameters involved in mineral metabolism and skeletal homeostasis. No detectable differences were observed in kidney histology between Mcu iKO and control mice at either 20 or 40 weeks of age (Supplementary Fig. 5a). In addition, urinary calcium and phosphate excretion, as well as serum levels of fibroblast growth factor 23, parathyroid hormone and vitamin D, were comparable between genotypes (Supplementary Fig. 5b,c).

### *Mcu* loss biases early mesenchymal lineage commitment

To determine whether *Mcu* deficiency alters early mesenchymal lineage decisions underlying the increased marrow adiposity observed in vivo, we performed fate-marker profiling at an early stage (day 3) in both C3H10T1/2 cells and primary bone marrow-derived cells. Under adipogenic differentiation conditions, *Mcu* knockdown markedly enhanced expression of adipogenic commitment markers, including *Zfp423*, *Pparg* and *Cebpd* (Supplementary Fig. 6a). By contrast, under osteogenic differentiation, *Mcu* knockdown suppressed early osteogenic commitment markers such as *Runx2*, *Sp7* and *Dlx5* (Supplementary Fig. 6b). Notably, fibrotic-related genes (*Col1a2*, *Acta2* and *Postn*) were modestly induced under osteogenic differentiation conditions following *Mcu* knockdown in primary BMCs, but not in C3H10T1/2 cells (Supplementary Fig. 6c). These findings suggest that Mcu deficiency preferentially biases early mesenchymal commitment toward adipogenesis, while fibrotic-like activation may occur in a context-dependent manner rather than representing a uniform fate outcome.

### *Mcu* regulates osteogenesis via both cell-autonomous and paracrine mechanisms

**Fig. 4:**
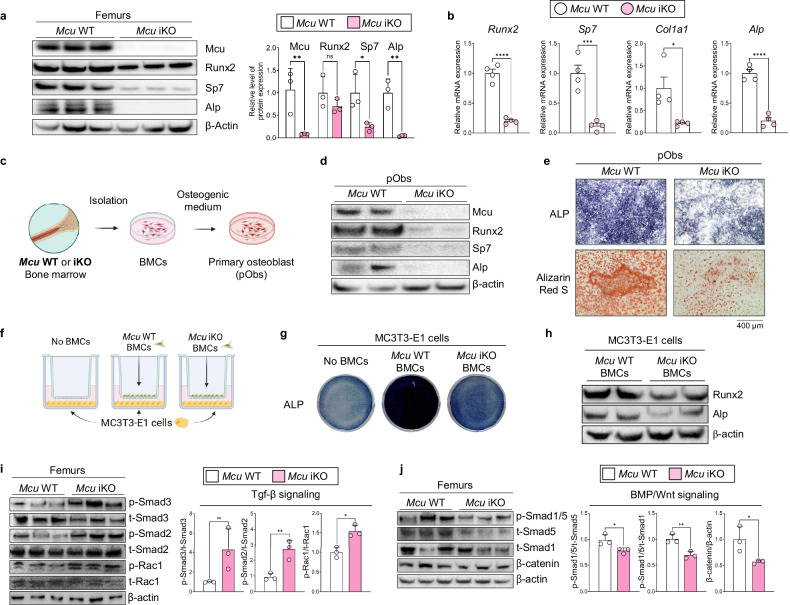
*Mcu* deficiency impairs osteogenesis through both cell-autonomous and paracrine mechanisms. **a** Immunoblot of mitochondrial and osteogenic proteins in femurs from *Mcu* WT and iKO mice. **b** qPCR analysis of osteogenic genes in femoral bone. **c** Experimental scheme: BMCs isolated from *Mcu* WT and iKO mice were differentiated into pObs. **d** Immunoblot of osteogenic markers in pObs. **e** ALP and Alizarin Red S staining of pObs. **f** Diagram of indirect trans-well culture; MC3T3-E1 cells in bottom well, BMCs in insert. **g** ALP staining of MC3T3-E1 cells after 7-day co-culture. **h** Osteogenic protein levels in MC3T3-E1 cells following co-culture. **i**, **j** Western blot analysis of TGF-β (**i**) and BMP/Wnt (**j**) signaling proteins. All experiments were conducted in mice aged 60 weeks. Data are presented as mean ± s.e.m. Statistical significance: *P < 0.05, **P < 0.01, ***P < 0.001, ****P < 0.0001.

To dissect the underlying cellular mechanisms, we next examined the intrinsic and extrinsic roles of Mcu in regulating osteoblast function and mesenchymal fate. We first assessed the expression of osteogenic markers in femoral bone tissues from aged *Mcu* iKO and control mice. The expression of Alp, Runx2 and Sp7 was significantly reduced in *Mcu* iKO femurs (Fig. 4a, b), indicating impaired osteogenic capacity in vivo. To determine whether this impairment was due to a cell-autonomous defect, we isolated BMCs from *Mcu* WT and iKO mice and induced osteogenically differentiated in vitro (Fig. 4c). *Mcu*-deficient pObs showed reduced expression of osteogenic genes (Fig. 4d and Supplementary Fig. 7a) and diminished matrix mineralization (Fig. 4e), supporting a cell-intrinsic requirement for Mcu in osteoblast differentiation. We next tested whether *Mcu*-deficient BMCs could influence the differentiation of neighboring osteoblasts through paracrine effects (Fig. 4f). MC3T3-E1 cell co-culture with *Mcu*-deficient BMCs exhibited markedly reduced ALP and Alizarin Red S staining (Fig. 4g and Supplementary Fig. 7b), along with decreased expression of osteogenic proteins (Fig. 4h). These results suggest that Mcu activity in BMCs contributes to a pro-osteogenic microenvironment, probably via secreted factors or altered metabolic signals.

Notably, the increased adipocyte accumulation observed in *Mcu* iKO bone marrow (Fig. 3h) suggested a shift toward adipogenic lineage allocation. To test this, BMCs from *Mcu* WT and iKO mice were subjected to adipogenic differentiation (Supplementary Fig. 7c). *Mcu*-deficient cells displayed increased lipid accumulation, as indicated by Oil Red O staining (Supplementary Fig. 7d). Mechanistically, western blot analysis revealed increased phosphorylation of Smad2/3 and decreased phosphorylation of Smad1/5 and β-catenin in *Mcu*-deficient femurs (Fig. 4i, j), indicating a shift from osteo-inductive BMP/Wnt signaling to Tgf-β-dominant signaling. These findings imply that *Mcu* is essential not only for intrinsic osteoblast differentiation but also for maintaining a microenvironment that supports bone formation and suppresses adipogenesis.

### *Mcu* maintains mitochondrial bioenergetics and dynamics during osteogenic differentiation

Given that osteoblast differentiation is an energy-intensive process and Mcu-mediated calcium signaling is critical for mitochondrial metabolism, we hypothesized that Mcu deficiency impairs osteogenesis by disrupting mitochondrial bioenergetics. To test this, we measured the OCR in MC3T3-E1 cells transfected with control or *Mcu*-specific siRNA. *Mcu* knockdown significantly reduced maximal respiration and ATP production (Supplementary Fig. 8), suggesting compromised OXPHOS during differentiation. These results indicate that impaired osteogenesis in *Mcu*-deficient cells may stem from insufficient mitochondrial ATP generation.

**Fig. 5:**
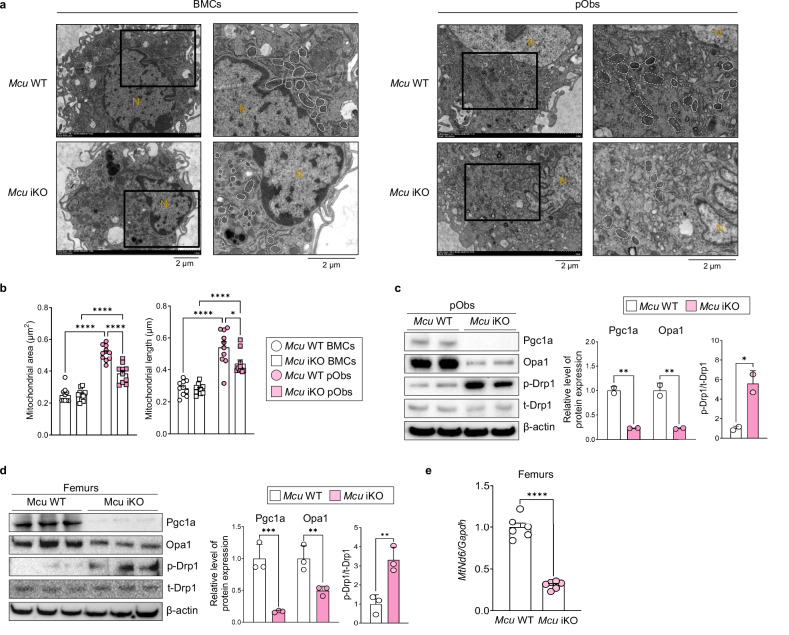
*Mcu* deficiency disrupts mitochondrial morphology and dynamics during osteoblast differentiation. **a** Transmission electron microscopy (TEM) images of BMCs and pObs from *Mcu* WT and iKO mice. Mitochondria are outlined with white; nuclei (N) are labeled in yellow. Black boxes indicate regions that are enlarged and shown on the right. **b** Quantification of mitochondrial area and length in TEM images. **c**, **d** Immunoblot of mitochondrial dynamics regulators in pObs (**c**) and femoral bone tissues (**d**). **e** qPCR of mitochondrial DNA copy number in femoral samples. Data are presented as mean ± s.e.m. Statistical significance: *P < 0.05, **P < 0.01, ***P < 0.001, ****P < 0.0001.

Because mitochondrial morphology is tightly linked to metabolic efficiency, we next examined mitochondrial dynamics during osteogenic differentiation. In control MC3T3-E1 cells, we observed a progressive elongation and expansion of mitochondria over time, consistent with enhanced fusion and network remodeling in response to increasing metabolic demands (Supplementary Fig. 9a). To assess whether this remodeling is disrupted by *Mcu* loss, we analyzed mitochondrial morphology in BMCs and pObs derived from *Mcu* iKO mice. Transmission electron microscopy revealed fragmented and shortened mitochondria in Mcu-deficient cells (Fig. 5a). Quantitative morphometry confirmed a significant decrease in mitochondrial area and length in (Fig. 5b and Supplementary Fig. 9b,c), indicating disrupted mitochondrial dynamics.

We next examined the molecular regulators of mitochondrial structure. Western blot analysis showed decreased expression of the mitochondrial fusion protein Opa1 and increased phosphorylation of Drp1, a key driver of mitochondrial fission, in *Mcu*-deficient pObs (Fig. 5c). Similar alterations were observed in femoral tissues from *Mcu* iKO mice (Fig. 5d), suggesting that the shift toward fission also occurs in vivo. In line with these structural changes, we detected a reduction in mitochondrial DNA content (Fig. 5e). These data demonstrate that Mcu is essential not only for maintaining mitochondrial function, but also for preserving mitochondrial integrity and abundance during osteoblast differentiation.

### Kaempferol enhances osteogenesis and mitochondrial function via Mcu-mediated calcium uptake

**Fig. 6:**
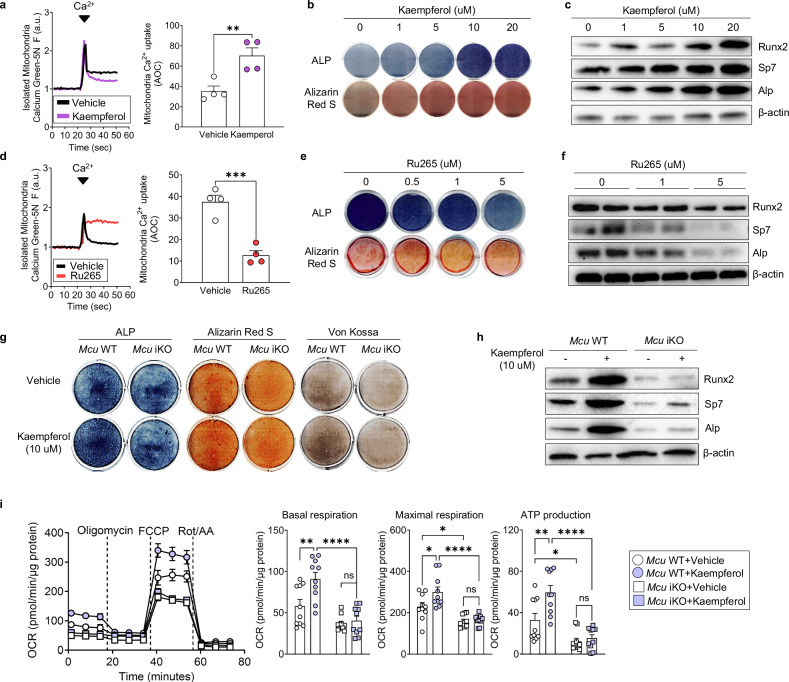
Kaempferol enhances osteogenesis and mitochondrial function via Mcu-mediated calcium uptake. **a** Mitochondrial calcium uptake in MC3T3-E1 cells treated with vehicle or kaempferol, assessed by Calcium Green-5N. **b** ALP (day 7) and Alizarin Red S (day 14) staining following kaempferol treatment. **c** Immunoblot of osteogenic markers post-treatment. **d** Calcium uptake following Ru265 treatment. **e** ALP and Alizarin Red S staining after Ru265. **f** Protein expression of osteogenic markers after Ru265 treatment. **g** Representative staining (ALP, Alizarin Red S, and von Kossa) staining of pObs from *Mcu* WT and iKO mice following kaempferol treatment. **h** Immunoblot of osteogenic markers in pObs. **i** OCR analysis of pObs after kaempferol treatment. Data are presented as mean ± s.e.m. Statistical significance: *P < 0.05, **P < 0.01, ***P < 0.001, ****P < 0.0001.

Given the central role of Mcu in osteoblast metabolism, we next asked whether pharmacological enhancement of mitochondrial calcium uptake could restore osteogenesis in *Mcu*-deficient settings. We treated MC3T3-E1 cells with kaempferol, a natural flavonoid previously shown to increase mitochondrial calcium uptake^23^. Kaempferol treatment significantly enhanced mitochondrial calcium uptake (Fig. 6a), which was accompanied by enhanced ALP activity and matrix mineralization in a dose-dependent manner (Fig. 6b). Protein levels of osteogenic markers were also upregulated (Fig. 6c), suggesting that kaempferol promotes osteogenic differentiation through mitochondrial activation. To test whether this effect is mediated via *Mcu*, we treated cells with Ru265, a Mcu inhibitor^24^. Ru265 treatment suppressed mitochondrial calcium uptake (Fig. 6d), reduced ALP and Alizarin Red S staining (Fig. 6e) and downregulated osteogenic protein expression (Fig. 6f), phenocopying *Mcu* knockdown. These results support the idea that Mcu activity is necessary for the osteogenic effects of kaempferol.

To further confirm the Mcu dependency of this response, we treated pObs derived from *Mcu* WT and iKO mice with kaempferol. Whereas kaempferol significantly enhanced mineralization and osteogenic protein expression in WT cells, it failed to do so in *Mcu*-deficient osteoblasts (Fig. 6g, h). This loss of responsiveness in the absence of *Mcu* demonstrates that the pro-osteogenic effects of kaempferol are strictly Mcu dependent.

Finally, we assessed whether kaempferol could also rescue mitochondrial respiration. Kaempferol treatment increased basal and maximal oxygen consumption, as well as ATP production, in WT cells but had no effect in *Mcu*-deficient cells (Fig. 6i), reinforcing the central role of mitochondrial calcium influx in osteoblast metabolism. Together, these findings indicate that kaempferol enhances both mitochondrial bioenergetics and osteoblast differentiation via Mcu-mediated calcium uptake, and thus may serve as a potential therapeutic strategy for bone loss disorders involving mitochondrial dysfunction.

## Discussion

In this study, we identify Mcu as a key regulator of osteoblast differentiation and bone integrity by modulating mitochondrial calcium uptake. We show that inducible knockout of *Mcu* in adult mice leads to impaired OXPHOS in osteoblast precursors, resulting in defective matrix formation and a skew toward adipogenic fate. These cellular defects translated into substantial bone loss in vivo, demonstrating that MCU-dependent calcium uptake is essential for bone formation. These results provide evidence that mitochondrial calcium handling directly regulates skeletal lineage allocation. These findings extend the emerging paradigm of ‘metabolic control of cell fate’ to bone biology, identifying mitochondrial calcium as a novel metabolic signal in osteogenesis^4^.

Mechanistically, loss of MCU blunted osteoblast energy metabolism. Mcu-deficient BMCs exhibited a dramatic reduction in oxygen consumption for ATP production, indicating that calcium-activated dehydrogenases in the TCA cycle were no longer fully engaged.

Moreover, we observed mitochondrial fragmentation with reduced expression of fusion protein (Opa1) and increased fission mediator (Drp1), suggesting a fission-dominant state. These mitochondrial changes probably reflect a stress response to energy deficiency, as oxidative stress or bioenergetic failure is known to induce Drp1-mediated fission and inhibit mitochondrial fusion^25^. By contrast, normal osteoblast differentiation requires robust mitochondrial function. A recent study showed that skeletal progenitors with genetically impaired complex I activity or OXPHOS cannot undergo osteogenesis^26^. Our results are consistent with a recent review highlighting that mitochondria in osteoblasts serve multiple roles—including ATP production, ROS buffering and secretion of mitochondrial signals—all geared toward supporting bone matrix synthesis^3^. Importantly, loss of Mcu not only disrupted osteoblast function intrinsically but also altered the extracellular niche. Co-culture experiments revealed that *Mcu*-deficient BMCs suppressed osteogenic differentiation of neighboring cells, suggesting impaired paracrine signaling or metabolic support. Taken together, *Mcu* deletion disrupts this mitochondrial program and deprives osteoblasts of the energy and redox milieu needed for differentiation.

Beyond metabolism, *Mcu* loss altered differentiation signaling. We found that *Mcu*-deficient BMCs upregulated adipogenic transcription factors and activated TGF-β signaling components, while downregulating BMP/Wnt signaling targets. This is reminiscent of the antagonist relationship between TGF-β and BMP/Wnt pathways in skeletal progenitors, where predominant TGF-β signaling suppresses osteogenic differentiation in favor of adipogenesis. Indeed, other groups have shown that elevated TGF-β signaling or reduced BMP/Wnt drives marrow adiposity and bone loss^27^. Our data suggest that mitochondrial calcium uptake normally restrains TGF-β activity during osteogenesis; when MCU is lost, unchecked Smad2/3 activity may push cells toward alternative fates. Importantly, fate-marker profiling under lineage-specific differentiation conditions revealed that *Mcu* deficiency enhanced adipogenic commitment in adipogenic environments, while suppressing osteogenic commitment even under osteogenic conditions. Under the latter condition, a modest and context-dependent induction of fibrotic-related genes was observed in primary BMCs, suggesting that fibrotic-like activation may reflect a secondary response to impaired osteogenic commitment rather than a primary fate decision. Thus, while fibrotic-related gene induction can be observed under osteogenic stress conditions, it does not appear to represent a dominant or primary lineage outcome of *Mcu* deficiency. The precise connection between mitochondrial calcium and these transcriptional programs remains to be defined. One possibility is that mitochondrial calcium modulates cytosolic calcium oscillations and downstream CaMK/NFAT pathways, which in turn cross-talk with BMP/Wnt^28^. Alternatively, changes in the NAD^+^/NADH ratio or mitochondrial ROS in *Mcu*-deficient cells might influence epigenetic regulators of lineage genes^29–31^. Notably, a recent study reported that *Mcu* overexpression in osteoblast-lineage cells inhibited BMP/Smad signaling via excessive ROS production, leading to impaired bone formation^32^. Together with our findings, this suggests that both deficient and excessive MCU activity disrupt osteogenesis, highlighting the importance of balanced mitochondrial calcium homeostasis.

Our work also explores translational angles. Kaempferol, a plant flavonol, has been reported to enhance mitochondrial calcium uptake and to promote osteogenic signaling^33^. In WT BMCs, we confirmed that kaempferol enhances mitochondrial calcium entry and boosts respiration and *Runx2* and *Sp7* expression. Strikingly, these effects were abrogated in *Mcu*-knockout cells, indicating that MCU is a necessary mediator of kaempferol’s pro-osteogenic action in our system. We also verified that MCU-specific inhibitor Ru265 similarly blocked osteogenesis, reinforcing this conclusion. Nonetheless, we caution that kaempferol has multiple cellular targets and antioxidant properties that may contribute to bone effect. Therefore, we interpret kaempferol as a proof-of-concept modulator of mitochondrial calcium, not as a selective MCU agonist. Although our data support an MCU-centered mechanism, future work must dissect these pathways. In addition, kaempferol’s poor bioavailability and off-target effects present challenges for clinical translation^34,35^. A related flavonoid, oleuropein, was recently shown to directly activate MCU and improve mitochondrial function in aged muscle^36^. Whether similar nutraceuticals could safely enhance bone formation remains an intriguing possibility.

Supporting the clinical relevance of our findings, we found that *MCU* expression levels decline in bone biopsies from postmenopausal women with osteoporosis and in ovariectomized mice, paralleling reports that aging and inflammatory signals downregulate MCU in other tissues^37^. This suggests that age-related bone loss may involve diminished mitochondrial calcium signaling. Thus, interventions that preserve or enhance MCU function could potentially mitigate skeletal aging. However, any such approach must consider safety: MCU is broadly expressed and required in heart, brain and muscle, so global modulation could have unintended effects. Targeted delivery to bone or use of cell-specific activators might be required. Moreover, our findings in mice need validation in human bone cells and careful dosing studies before clinical application.

Several limitations of this study should be noted. First, the use of a whole-body inducible knockout model does not distinguish between cell-autonomous and systemic effects. Although inducible model avoids developmental confounders and in vitro differentiation and co-culture assays support an osteoblast-intrinsic role for Mcu, we cannot rule out contributions from other tissues. For example, Mcu deletion in osteoclast or hematopoietic cells could indirectly affect bone remodeling, and systemic metabolic disturbances in muscle or endocrine organs might influence bone formation. To address these concerns, we carefully evaluated major renal and endocrine parameters that are known to secondarily influence skeletal homeostasis. Notably, *Mcu* iKO mice did not exhibit detectable abnormalities in kidney histology, urinary calcium or phosphate excretion, or circulating levels of FGF23, parathyroid hormone or vitamin D. These findings suggest that overt disruptions in classical renal–endocrine mineral homeostasis are unlikely to account for the bone loss and marrow adiposity observed in *Mcu* iKO mice. Nevertheless, more subtle systemic or interorgan effects cannot be excluded, and future studies using osteoblast- or lineage-specific Cre drivers will be required to definitively resolve cell-autonomous versus systemic contributions of MCU to bone biology. Second, while we observe consistent changes in mitochondrial morphology and function, it remains to be tested whether these alterations impact chromatin remodeling or epigenetic control of osteogenesis, given the known role of mitochondria-derived metabolites in regulating gene expression^38^. Third, our study did not fully characterize adipogenesis versus fibrosis, although we observed increased marrow fat histologically. Follow-up lineage-tracing and adipocyte-specific analyses would clarify whether Mcu-deficient mesenchymal progenitors preferentially adopt an adipocyte fate or generate fibrotic marrow stroma under stress.

In conclusion, our findings highlight Mcu as a central regulator of osteoblast bioenergetic, mesenchymal lineage commitment and bone integrity. By linking mitochondrial calcium uptake to osteogenic versus adipogenic fate decisions, we uncover a new dimension in bone biology that integrates metabolism and signaling. Future investigations into molecular mechanisms downstream of MCU, and the development of bone-targeted mitochondrial modulators, may open novel therapeutic avenues for osteoporosis and age-related fracture risk.

## Supplementary information

Supplementary Information
