# Supplementary material for: Mcu regulates bone formation via mitochondrial calcium uptake and lineage allocation
Source: Exp Mol Med. 2026 May 1;58(5):1439–48. doi: 10.1038/s12276-026-01705-3 (PMC13234314; doi:10.1038/s12276-026-01705-3)
Supplement: Supplementary file 1 — Supplementary Information [file 12276_2026_1705_MOESM1_ESM.pdf]

Supplementary Figures

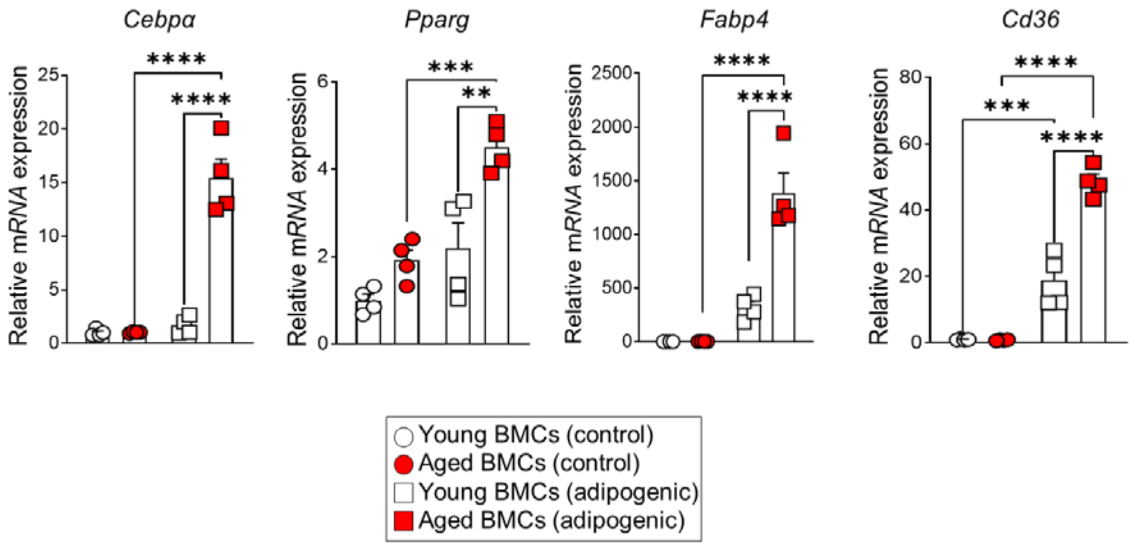

Supplementary Fig. 1 Increased adipogenic marker expression in aged bone marrow cells. qPCR analysis of adipogenic genes in bone marrow cells (BMCs) isolated from young and aged mice after 5 days of adipogenic induction. Data are presented as mean ± SEM. Statistical significance: ***P* < 0.01, ****P* < 0.001, *****P* < 0.0001.

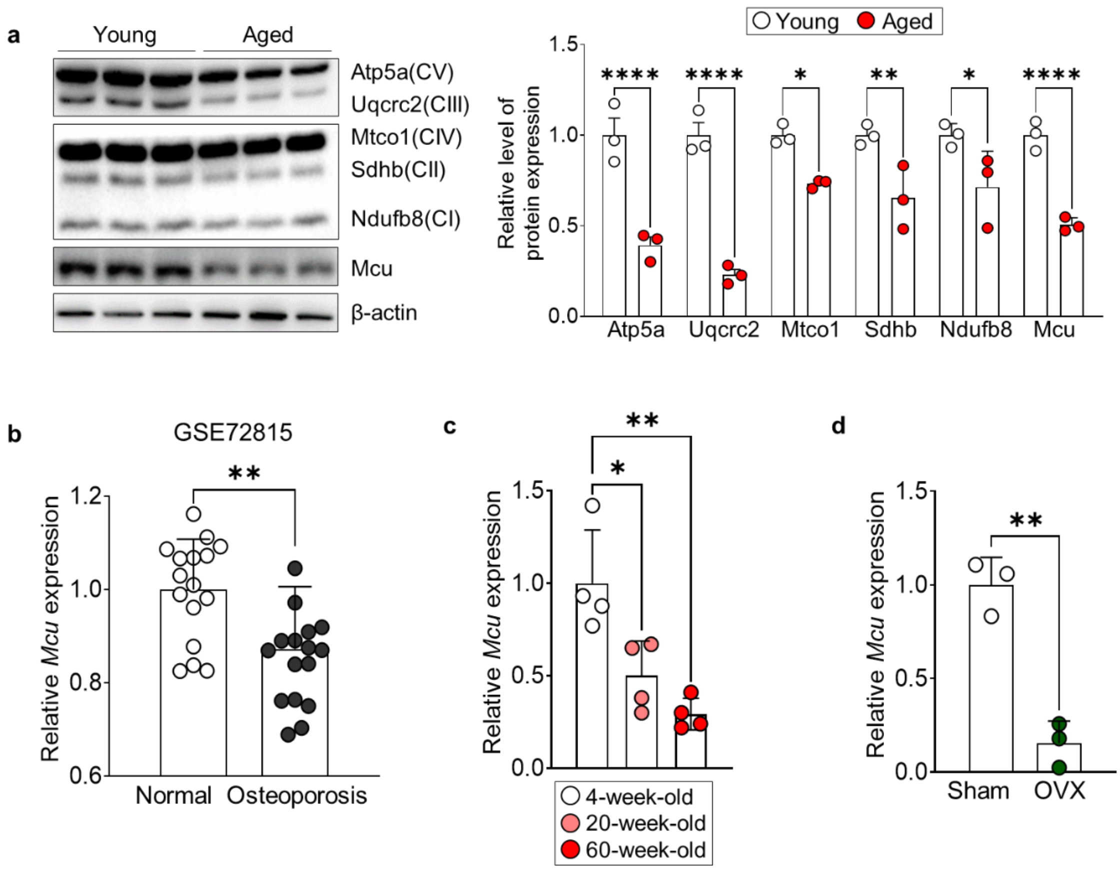

Supplementary Fig. 2 Age-associated reduction in Mcu expression. a Immunoblot analysis of Mcu and OXPHOS subunits in primary osteoblasts derived from young and aged mice. **b** *MCU* mRNA expression from iliac crest bone biopsies in old women (GSE72815). **c** qPCR analysis of *Mcu* expression in femurs from 4- and 20-week-old mice. **d** *Mcu* expression in femoral bone tissues from sham-operated and ovariectomized (OVX) mice. Data are presented as mean ± SEM. Statistical significance: *$P < 0.05$, **$P < 0.01$, ****$P < 0.0001$.

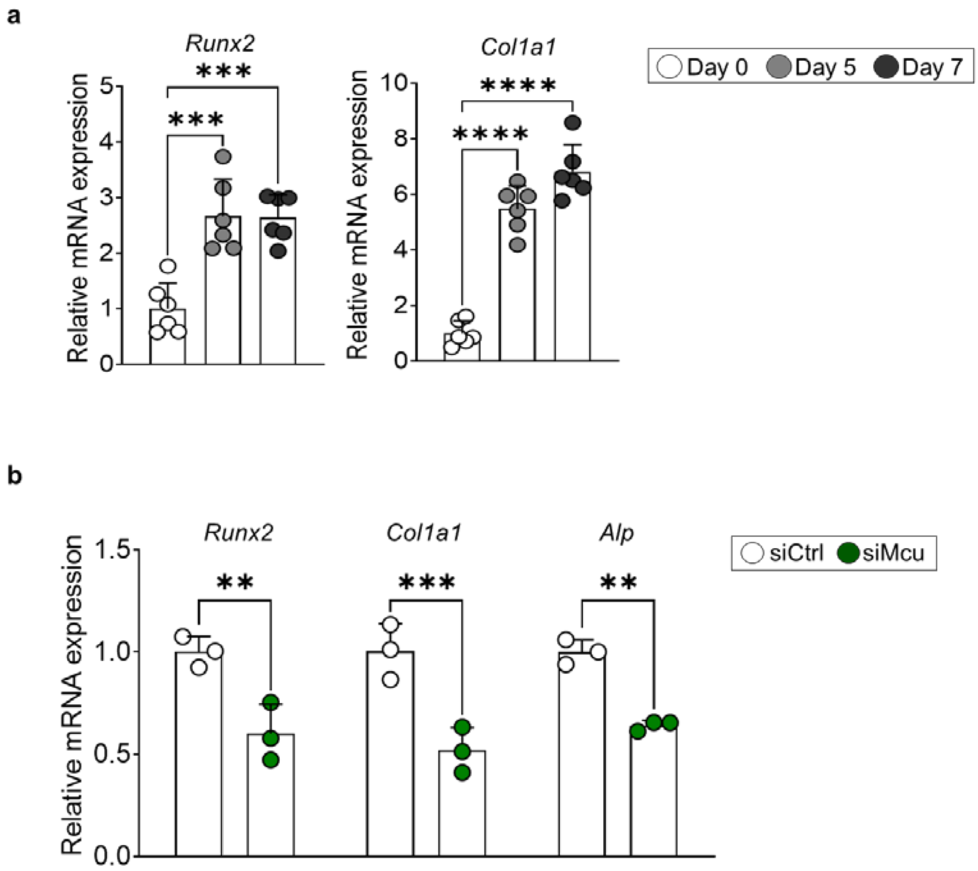

Supplementary Fig. 3 Temporal induction of osteogenic genes and Mcu during differentiation. a mRNA expression levels of osteogenic marker during differentiation of MC3T3-E1 cells. **b** mRNA expression of osteogenic genes in MC3T3-E1 cells transfected with siCtrl and siMcu cells after 7 days of differentiation. Data are presented as mean ± SEM. Statistical significance: **$P < 0.01$, ***$P < 0.001$, ****$P < 0.0001$.

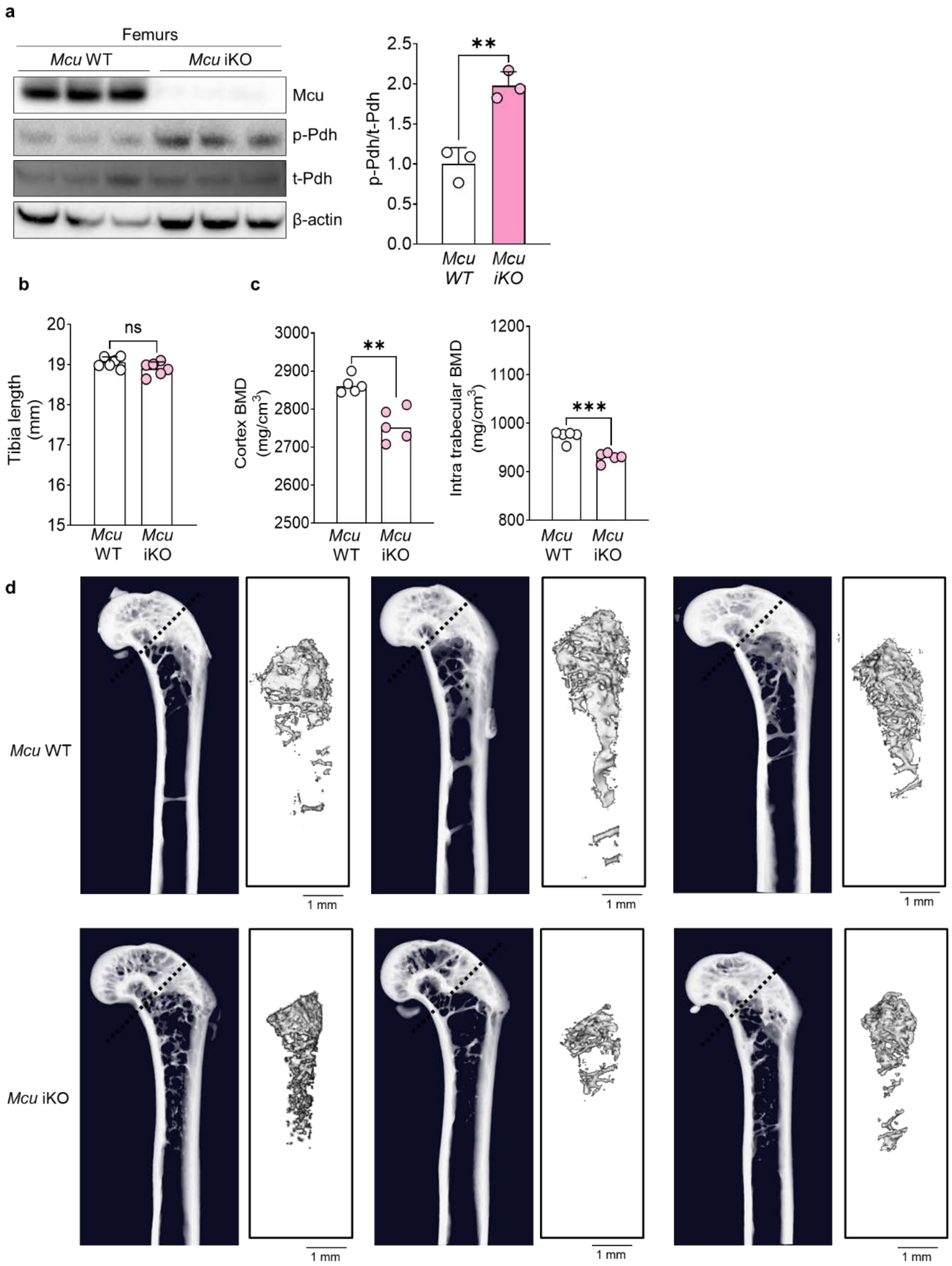

Supplementary Fig. 4 Skeletal phenotypes in *Mcu* iKO mice. a Western blot analysis of phosphorylated and total Pdh in femur tissues from WT and *Mcu* iKO mice. **b** Measurement of tibia length at 10 months. **c** Cortical and trabecular bone marrow density. **d** 3D reconstruction of trabecular regions from µCT analysis. Data are presented as mean ± SEM. Statistical significance: $^{**}P < 0.01$, $^{***}P < 0.001$.

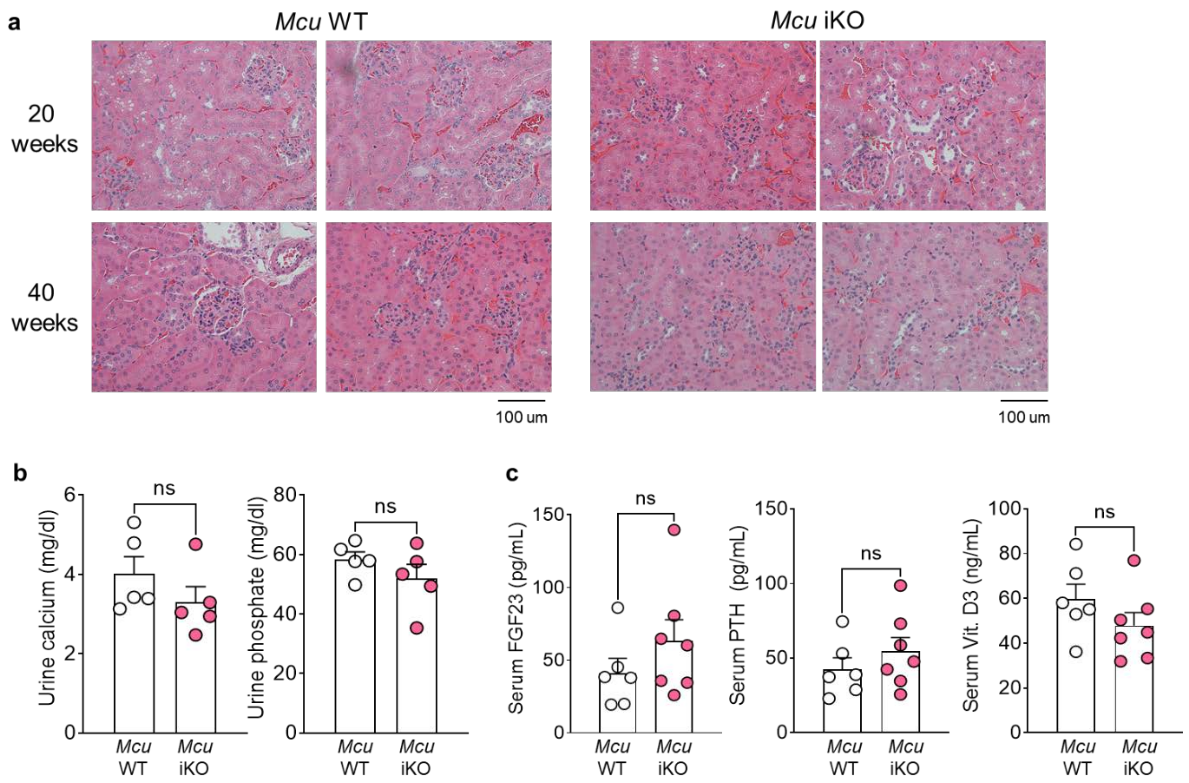

Supplementary Fig. 5 Assessment of renal and systemic mineral homeostasis in Mcu iKO mice. **a** Representative H&E staining of kidneys from 20- and 40-week-old mice of WT and *Mcu* iKO mice. **b** Quantification of urinary calcium and phosphate levels. **c** Serum levels of fibroblast growth factor 23 (FGF23), parathyroid hormone (PTH), and vitamin D3 measured in 40-week-old mice. Data are presented as mean ± SEM.

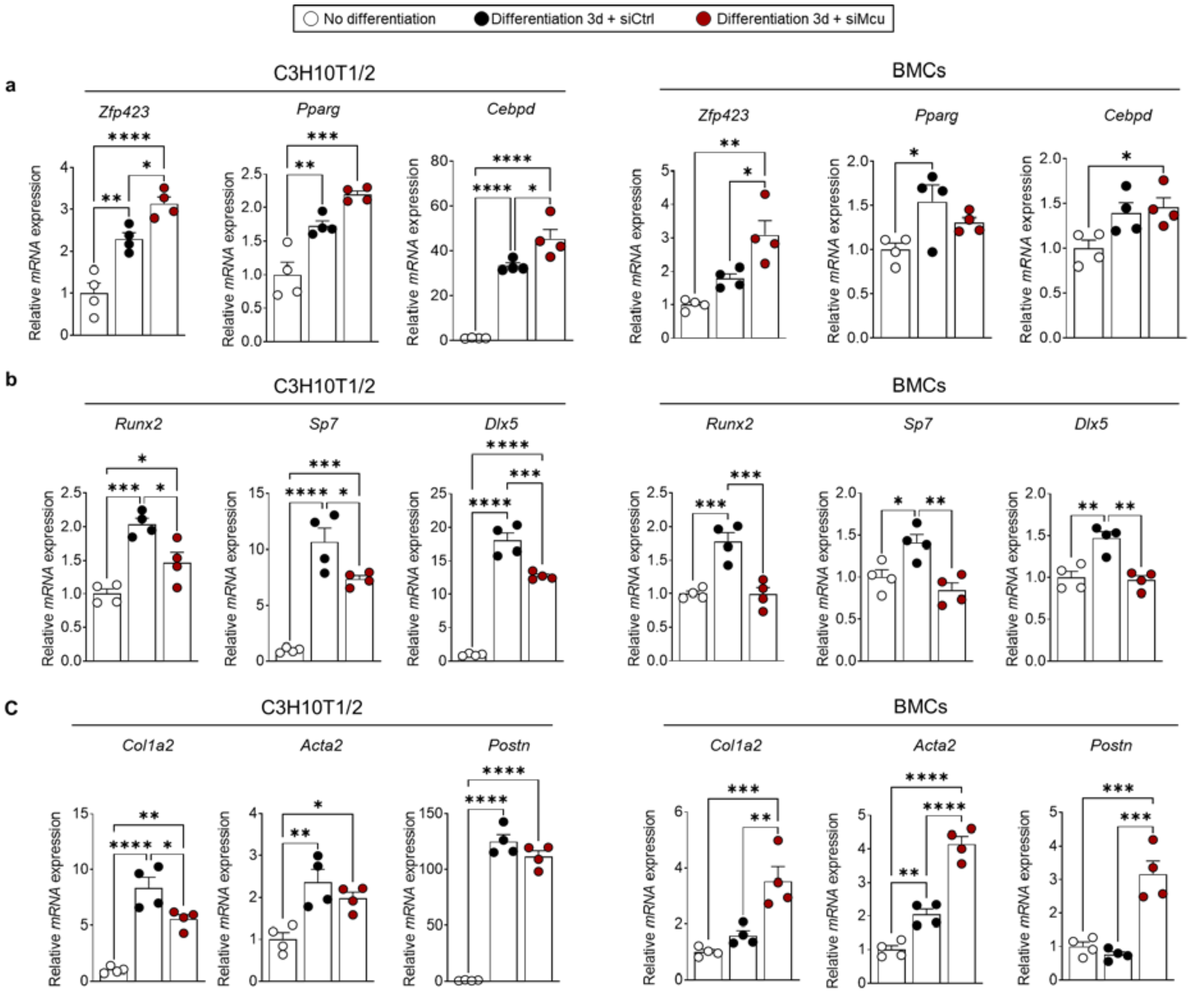

Supplementary Fig. 6 Mcu deficiency biases early mesenchymal lineage commitment toward adipogenesis. Quantitative PCR analysis of early fate-associated gene expression in C3H10T1/2 cells and primary bone marrow-derived cells (BMCs) under lineage-specific differentiation conditions. Cells were analyzed under undifferentiated conditions or after 3 days of differentiation following transfection with control or *Mcu*-targeting siRNA. **a** Adipogenic commitment markers under adipogenic differentiation conditions. **(b, c)** Osteogenic commitment markers (**b**) and fibrotic-related genes (**c**) under osteogenic differentiation conditions. Data are presented as mean ± SEM. Statistical significance: *$P < 0.05$, **$P < 0.01$, ***$P < 0.001$, ****$P < 0.0001$.

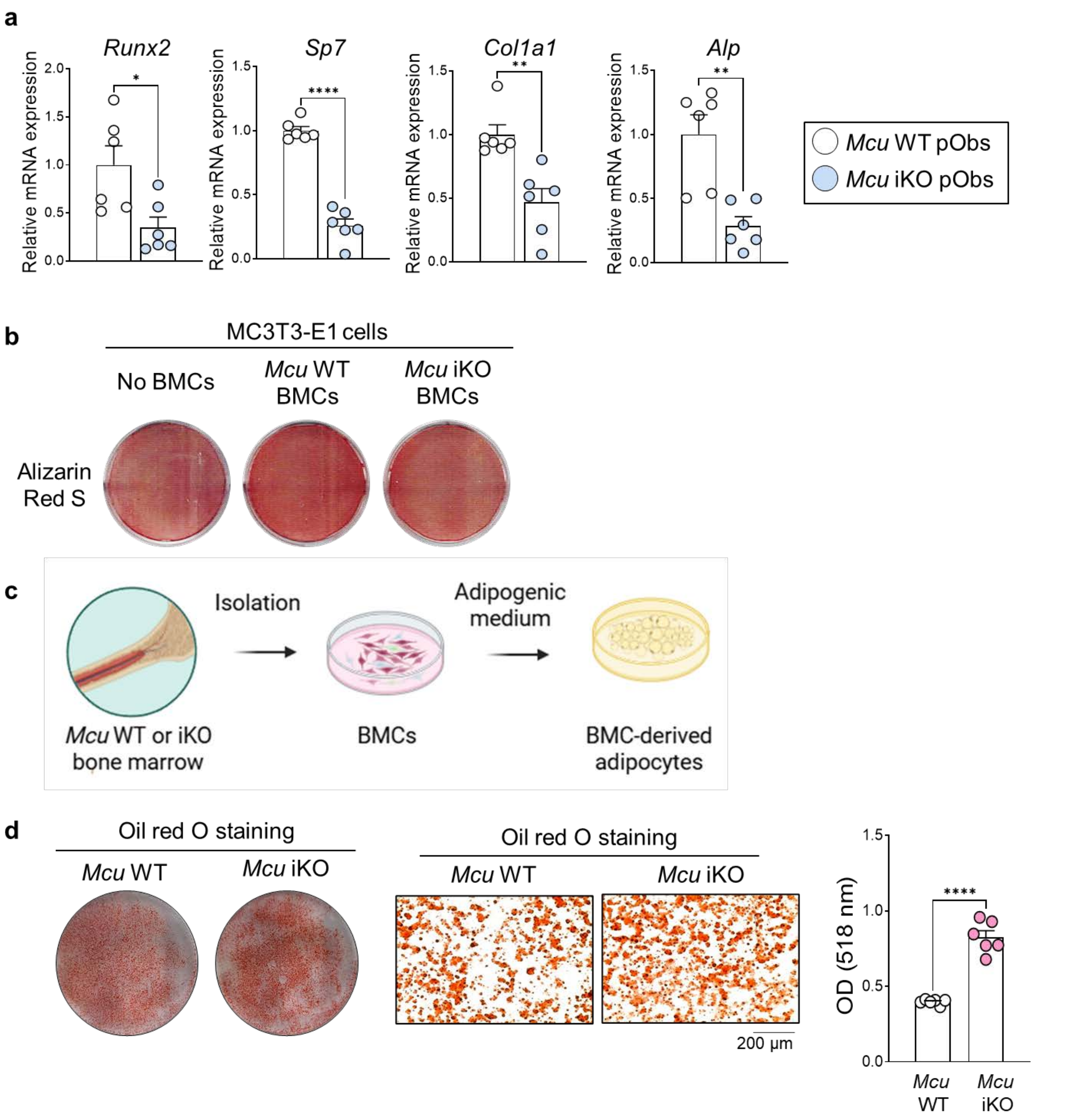

Supplementary Fig. 7 Cell-autonomous and paracrine defects in osteogenesis and increased adipogenesis in *Mcu*-deficient BMCs. a mRNA expression of osteogenic genes in primary osteoblasts isolated from *Mcu* WT and iKO mice. **b** Mineralization of MC3T3-E1 cells co-cultured with *Mcu* WT or iKO BMCs using a transwell co-culture system. **c** Schematic illustration of the adipogenic induction of BMCs. **d** Oil Red O staining of BMC-derived adipocytes isolated from *Mcu* WT and iKO mice.

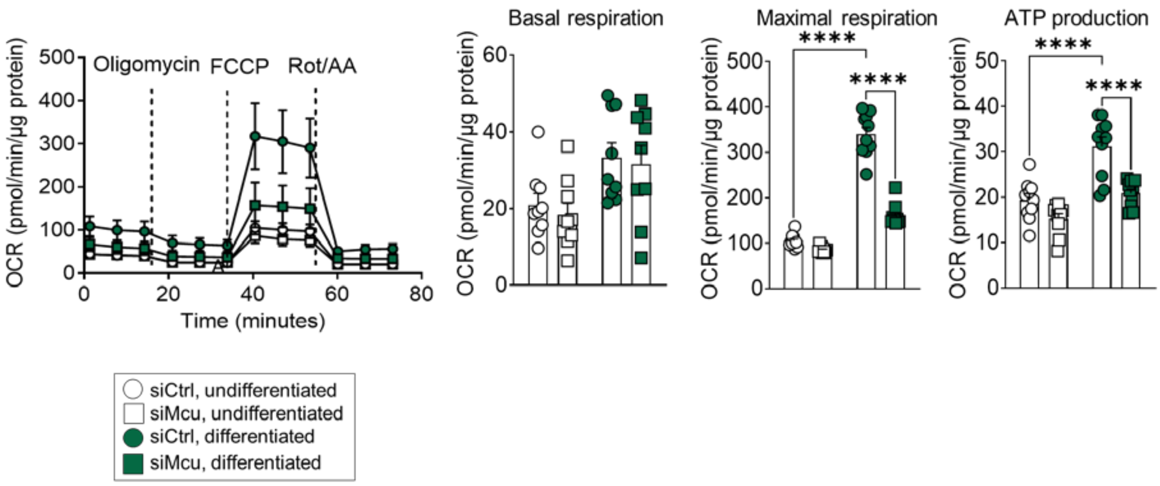

Supplementary Fig. 8 Mcu is required for mitochondrial respiration during osteogenic differentiation. Oxygen consumption rate analysis in undifferentiated and differentiated MC3T3-E1 cells with siCtrl or siMcu. Data are presented as mean ± SEM. Statistical significance: ****P < 0.0001.

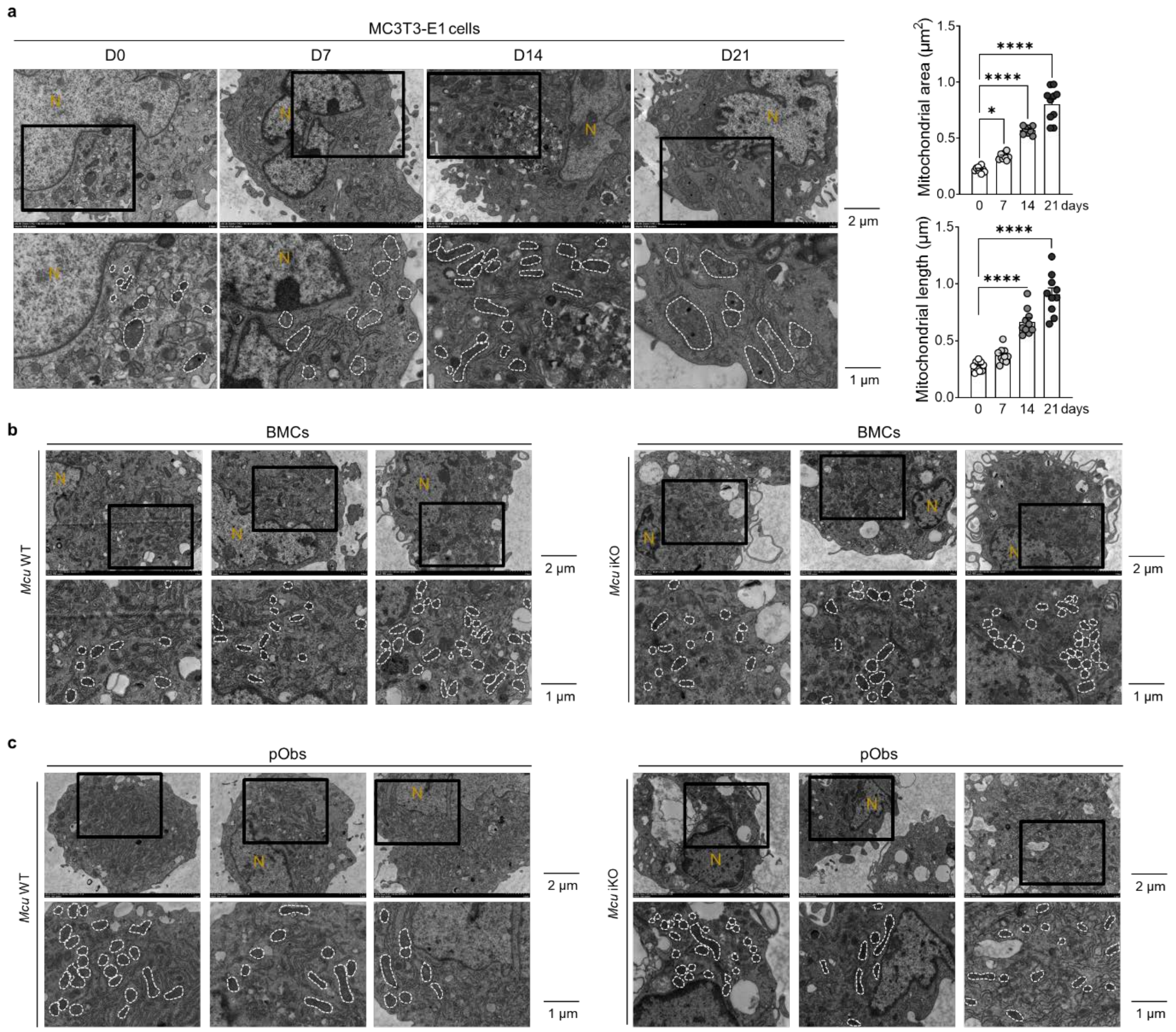

Supplementary Fig. 9 Mcu deficiency impairs mitochondrial network remodeling during differentiation. a Mitochondrial morphology and quantification (area and length) during the differentiation period of MC3T3-E1 cells. **b, c** Additional TEM images corresponding to Fig. 5A. Black boxes indicate the regions selected for high-magnification views. Mitochondria are outlined with white dashed lines. Data are presented as mean ± SEM. Statistical significance: *P < 0.05, ****P < 0.0001.

Supplementary Tables

Supplementary Table 1. Primer sequences used in this study

Gene	Forward	Reverse
Runx2	TGTGGCTGTTGTGATGCGTA	GGCTACAACCTTGAAGGCCA
Sp7	GAACAAGAGTGAGCTGGCCT	AGCTTCTTCCTGGGTAGGCT
Col1a1	AGACGGGAGTTTCTCCTCGG	TGTAGACTCTTTGCGGCTGG
Alp	GGTGGTCACAGCAGTTGGTA	GACGTTCCGATCCTGAGTGG
Mcu	CGCCAGGAATATGTTTATCCA	CTTGTAATGGGTCTCTCAGTCTCTT
Cebpa	TGCGCAAGAGCCGAGATAAA	TCACTGGTCAACTCCAGCAC
Pparg	CCATTCTGGCCCACCAAC	AATGCGAGTGGTCTTCCATCA
Fabp4	CACCGCAGACGACAGGAAG	GCACCTGCACCAGGGC
Cd36	GGCCAAGCTATTGCGACAT	CAGATCCGAACACAGCGTAGA
Rplp0	TCGTTGGAGTGACATCGTCT	TAGTTGGACTTCCAGGTCGC
Zfp423	GCGCATCTTGCCTCAAAGAG	ACATTGCAGCTTGAACAGCG
Cebpd5	CAAGCTGAGCGACGAGTACA	AGCTGCTCCACCTTCTTCTG
Dlx5	AGGCTTATGCCGACTACGGCTA	CTCTGGCTCCGCCACTTCTTTC
Col1a2	CCAGAGTGGAACAGCGATTAC	GATGCAGGTTTCACCAGTAGAG
Acta2	GTCCCAGACATCAGGGAGTAA	TCGGATACTTCAGCGTCAGGA
Postn	CCTGCCCTTATATGCTCTGCT	AAACATGGTCAATAGGCATCACT
Mt-Nd6	AGGTGAAGGCTTTAATGCTAACCC	GGTCGCAGTTGAATGCTGTGT
Gapdh	GAACTCCTCATGGGTCTGTAGTG	TGTTGTGGTACGTGCATAGCTG

Supplementary Table 2. List of antibodies

Antibodies	Source	Identifier
Runx2	Santa Cruz	Sc-390351
Sp7	Santa Cruz	Sc-393325
Alp	Santa Cruz	Sc-271431
β-actin	Santa Cruz	Sc-69879
Mcu	Cell signaling	14997S
Atp5A	Abcam	ab14748
OXPHOS cocktail (Atf5a, Uqcrc2, MtCo1, Sdhb, Ndufb8)	Abcam	ab110413
p-Smad3	Cell signaling	9520
t-Smad3	Cell signaling	9523
p-Smad2	Cell signaling	5339
t-Smad2	Cell signaling	3108
p-Rac1	Thermo Fisher Scientific	PA5-104640
t-Rac1	Thermo Fisher Scientific	PA1-091X
p-Smad1/5	Cell signaling	9516
t-Smad1	Cell signaling	9743
t-Smad5	Cell signaling	9517
b-Catenin	Cell signaling	9562
Pgc1α	Abcam	ab191838

Opa1	BD Biosciences	BD 612606
p-Drp1	Thermo Fisher Scientific	PA5-64821
t-Drp1	Abcam	ab184247
p-Pdh, Ser293	Sigma	ABS204
t-Pdh	Invitrogen	459400
